# How should HIV resources be allocated? Lessons learnt from applying Optima HIV in 23 countries

**DOI:** 10.1002/jia2.25097

**Published:** 2018-04-13

**Authors:** Robyn M Stuart, Laura Grobicki, Hassan Haghparast‐Bidgoli, Jasmina Panovska‐Griffiths, Jolene Skordis, Olivia Keiser, Janne Estill, Zofia Baranczuk, Sherrie L Kelly, Iyanoosh Reporter, David J Kedziora, Andrew J Shattock, Janka Petravic, S Azfar Hussain, Kelsey L Grantham, Richard T Gray, Xiao F Yap, Rowan Martin‐Hughes, Clemens J Benedikt, Nicole Fraser‐Hurt, Emiko Masaki, David J Wilson, Marelize Gorgens, Elizabeth Mziray, Nejma Cheikh, Zara Shubber, Cliff C Kerr, David P Wilson

**Affiliations:** ^1^ Department of Mathematical Sciences University of Copenhagen Copenhagen Denmark; ^2^ Burnet Institute Melbourne VIC Australia; ^3^ Institute for Global Health University College London London UK; ^4^ Clinical Operational Research Unit Department of Mathematics University College London London UK; ^5^ Department of Applied Health Research University College London London UK; ^6^ Department of Global Health and Development Faculty of Public Health and Policy London School of Hygiene & Tropical Medicine London UK; ^7^ Institute of Global Health University of Geneva Geneva Switzerland; ^8^ Institute of Social and Preventive Medicine University of Bern Bern Switzerland; ^9^ Institute of Mathematical Statistics and Actuarial Science University of Bern Bern Switzerland; ^10^ Institute of Mathematics University of Zurich Zurich Switzerland; ^11^ School of Public Health and Preventive Medicine Monash University Melbourne VIC Australia; ^12^ School of Physics University of Sydney Sydney NSW Australia; ^13^ The Kirby Institute UNSW Sydney Sydney NSW Australia; ^14^ The World Bank Group Washington DC USA

**Keywords:** HIV modeling, allocative efficiency, cost‐effectiveness, optimal HIV investment, resource allocation, resource needs

## Abstract

**Introduction:**

With limited funds available, meeting global health targets requires countries to both mobilize and prioritize their health spending. Within this context, countries have recognized the importance of allocating funds for HIV as efficiently as possible to maximize impact. Over the past six years, the governments of 23 countries in Africa, Asia, Eastern Europe and Latin America have used the Optima HIV tool to estimate the optimal allocation of HIV resources.

**Methods:**

Each study commenced with a request by the national government for technical assistance in conducting an HIV allocative efficiency study using Optima HIV. Each study team validated the required data, calibrated the Optima HIV epidemic model to produce HIV epidemic projections, agreed on cost functions for interventions, and used the model to calculate the optimal allocation of available funds to best address national strategic plan targets. From a review and analysis of these 23 country studies, we extract common themes around the optimal allocation of HIV funding in different epidemiological contexts.

**Results and discussion:**

The optimal distribution of HIV resources depends on the amount of funding available and the characteristics of each country's epidemic, response and targets. Universally, the modelling results indicated that scaling up treatment coverage is an efficient use of resources. There is scope for efficiency gains by targeting the HIV response towards the populations and geographical regions where HIV incidence is highest. Across a range of countries, the model results indicate that a more efficient allocation of HIV resources could reduce cumulative new HIV infections by an average of 18% over the years to 2020 and 25% over the years to 2030, along with an approximately 25% reduction in deaths for both timelines. However, in most countries this would still not be sufficient to meet the targets of the national strategic plan, with modelling results indicating that budget increases of up to 185% would be required.

**Conclusions:**

Greater epidemiological impact would be possible through better targeting of existing resources, but additional resources would still be required to meet targets. Allocative efficiency models have proven valuable in improving the HIV planning and budgeting process.

## 1 Introduction

If decisions on the allocation of health resources were guided by the principals of health economics alone, funds would be allocated in ways intended to lead to the greatest reductions in disease burden overall. However, economics is not – and has never been – the sole factor influencing decisions on the allocation of health funds. Such decisions are also influenced by numerous other important factors, including the desires of different funding bodies, the influence of other sectors, historical precedent, the desire to promote equity among vulnerable or prioritized population groups, attempts to reduce financial risk and the desire to maintain health security. In addition, governments and other health funders are often challenged by a lack of information about the cost‐effectiveness and impact of health interventions at a population level. The combination of these factors means that the allocation of health funds is often vastly different to how it would be under a purely population‐level, evidence‐based and health‐outcome‐focused framework 1.

As with any component of health budgeting, planning an HIV response can be an extremely time‐consuming process. To aid with this process, several different tools have been developed and employed in different contexts, including the widely used GOALS resource estimation tool, the Asian Epidemic model and the Optima HIV tool 2. All three tools are equipped with a resource requirements estimation feature intended to help with budgeting. However, a unique feature of the Optima HIV tool is its allocative efficiency optimization algorithm. Within health economics, a response is described as allocatively efficient if funds are allocated across different HIV interventions and delivery modalities in the way that leads to the best possible epidemic outcomes given any relevant constraints. This is particularly important in the current epidemiological 3 and funding 4, 5 context. Since 2002, an estimated US$80.3 billion in development assistance for HIV programmes has been disbursed in over 100 lower‐income countries 6. However, the trend in funding over the past seven years has been almost flat. Development assistance for HIV in 2015 totalled US$7.5 billion, which represented a 13% decrease from 2014 levels (the first funding decrease in five years) and brought the total amount of funding back to 2008 levels 6. Thus, the question of how to get the most out of the available HIV funding is more essential now than ever before. It is now generally accepted that resource allocation decisions should be informed by, or grounded in, explicit criteria based on cost‐effectiveness to maximize health benefits with the resources available 7, 8, 9, 10, 11, 12, 13, 14, 15, 16, 17, 18, 19, 20.

In this paper, we discuss and compare studies conducted over six years and across 23 countries in Africa, Asia, Eastern Europe and Latin America, each of which used Optima HIV to estimate the potential gains that could be achieved by reallocating resources in a more efficient way. We do not intend to provide a formal meta‐analysis, but rather a broad qualitative comparison of the results that the modelling analyses found in each context. By synthesizing the results, we aim to identify common principles for the optimal allocation of HIV resources.

## 2 Methods

The 23 studies included in this review were conducted in Indonesia 21 and Vietnam 22 from the East Asia and Pacific (EAP) region; Argentina 23, Colombia 24, Mexico 25, and Peru 26 from the Latin America and Caribbean (LAC) region; Armenia 27, Belarus 28, Bulgaria 29, Georgia 30, Kazakhstan 31, Kyrgyzstan 32, Macedonia 33, Moldova 34, Tajikistan 35, Ukraine 36, and Uzbekistan 37 from the Eastern Europe and Central Asia (EECA) region; Zambia 38 from the Sub‐Saharan Africa (SSA) region; and Cote d'Ivoire 39, Niger 40, Senegal 41, Sudan 42, and Togo 43 from the West and Central Africa (WCA) region. Studies were conducted in partnership with institutions including the World Bank, the Global Fund to fight AIDS, Tuberculosis and Malaria (the Global Fund), the United States President's Emergency Plan for AIDS Relief (PEPFAR), the HIV Modelling Consortium, the Joint United Nations Programme on HIV/AIDS (UNAIDS) and the United Nations Development Programme (UNDP).

Although there was variation in the populations and programmes that were considered across the 23 studies, our focus is on the similarities that emerge from applying the same methodological framework for the analysis of the efficiency of the HIV response. Thus, the criteria for inclusion in this review were: (1) that the study was requested by the government of the country in question; (2) that the Optima HIV model was used to estimate the mathematically optimal distribution of national HIV resources given government‐specified epidemiological targets; (3) that the analysis represented the entire country's epidemic; (4) that the government agreed for results to be released; and (5) that the study had not been replaced by a more recent study that was already being included. The first three criteria were applied in order to ensure comparability of results, the fourth was added because some of the studies were confidential and intended solely for internal ministry purposes, and the fifth was added to avoid redundancy. The 23 studies mentioned previously comprise the full quota of studies that met these criteria. The Optima HIV model was also used in other country studies, and in analyses at sub‐national regions, but these additional studies did not meet the above criteria and thus were not included in this review (see Table S1 for a complete listing).

The Optima HIV tool was designed and developed by the Optima Consortium for Decision Science (the Optima Consortium) with technical inputs and guidance from the World Bank. The tool itself is based on a compartmental model of HIV transmission and disease progression, and is capable of producing estimates of epidemic trends, resource needs, and the impact and cost‐effectiveness of HIV responses. Furthermore, it can estimate the allocation of resources across programmes that best addresses national HIV targets while considering various logistic, political and ethical constraints 44.

Each study commenced with a request, made to a development agency by the national government, for technical assistance in conducting an HIV allocative efficiency study. In most cases, this request was made to the World Bank; in two cases (Tajikistan and Uzbekistan) it was made to the UNDP; and in six cases (Armenia, Belarus, Kazakhstan, Kyrgyzstan, Moldova and Ukraine), it was made to a group of funding agencies. The agreement to conduct a study using Optima HIV was then formalized on the basis of a scope of work document that outlined the key policy questions for the modelling analysis. An analytic team was then formed to carry out the work agreed upon, with team members typically including representatives from the government (e.g. from the ministry of health, the national team responsible for monitoring and evaluation, or the national AIDS commission), from partnering organizations (e.g. the World Bank, the Global Fund, UNDP, PEPFAR, UNAIDS) and from the Optima Consortium. The analytic team then proceeded to follow the steps outlined in Table 1. Reports with full details of data, context, methods, results, interpretation and discussion for all 23 countries are available either on the Optima Consortium website (http://www.ocds.co) or the World Bank's Open Knowledge Repository, or can be made available upon request.

**Table 1:** Steps in an allocative efficiency study, as followed for each of the countries considered in this review

Step	Rationale	Processes followed	Difficulties encountered and steps taken to overcome them
1. Identify the population groups and HIV programmes suitable for inclusion in the analysis.	The burden of HIV varies considerably within countries according to factors such as geography, behavioural tendencies, age and sex. The population groups included in an allocative efficiency study should be selected to capture this heterogeneity.	In all 23 studies, the entire national population was stratified according to age, sex and risk behaviour. In addition, the population was further stratified according to geographical region in Moldova and Cote d'Ivoire.	The desire to capture the particulars of the epidemic dynamics must be weighed up against the practical constraints around data availability. Criteria were defined to guide the decision on whether to include a population: the population should (a) be clearly defined, (b) play a substantial role in the country's epidemic, (c) currently or could be targeted with HIV programmes, and (d) have a minimum amount of data or reliable estimates on population size and HIV prevalence.
2. Collect and validate the data required for the analysis.	A determination of how to optimally target an HIV response must be data‐driven. Demographic, behavioural and epidemiological data for each population group must be collected, as well as programmatic data including unit costs, expenditure and historical levels of coverage (particularly important for antiretroviral therapy programmes) for each programme and service delivery modality.	All available data were collected and validated by the analytic teams.	In several contexts, there were data gaps in the epidemiological, behavioural and programmatic data. Often, this step and the first step were conducted iteratively, with populations being first considered for inclusion and then later removed if insufficient data were available.
3. Calibrate the model to available data.	The calibration process involves adjusting a subset of the model's parameters in order to minimize the mean absolute percentage error between the model's estimates and the observed data, and then subjecting the projections produced by the model to a process of scrutiny and validation by the district, province and national health departments.	Typically, the model was calibrated to historical data on HIV prevalence, the number of HIV diagnoses, and the number of people receiving antiretroviral therapy, as well as (where requested) the outputs of other models that the country had previously used.	Attaining a realistic calibration relies on having good data to input to the model. When difficulties were experienced with calibrations, this would often indicate issues with the underlying data. In this sense, the process of model calibration is conducted synchronously with the process of data validation.
4. Establish cost functions.	Cost functions define a relationship between spending on an HIV service and the expected coverage and outcome of that service amongst the target population.	Each analytic team agreed on realistic assumptions on both the maximal attainable coverage for each programme/modality and the behavioural outcome expected to prevail under that maximal coverage level.	Data to inform cost functions is difficult to obtain. In most cases, the cost functions were partially informed by data and partially be expert opinion.
5. Calculate the optimal allocation of available funds.	The allocation of funds that would deliver the outcome closest to national strategic targets can be calculated using Optima's mathematical optimization algorithm.	National strategic targets were identified by the analytic teams, usually in consultation with ministries of health or other responsible bodies.	In some cases, the initial optimization produced a recommendation that the country deemed politically or programmatically infeasible. In such cases, there was an option to rerun the optimization with additional constraints.
6. Produce epidemic trajectories.	The future evolution of the HIV epidemic depends on the future of the HIV response. The previous analytic steps defined the nature of this dependency, and determined the response that would lead to the best epidemic outcomes. The final step translates these responses into epidemic outcomes.	We projected the future evolution of the epidemic assuming that the future HIV budget was allocated (i) as per the last reported HIV spending pattern and (ii) as per the optimal allocation of funds calculated in the previous step.	The future of HIV funding is uncertain. To account for this uncertainty, epidemic projections were produced under a range of different assumptions about future budget availability.

In this review, we analyze the findings across the 23 studies using a thematic analysis, stipulating that these themes must have been a conclusion of at least three studies before they could be included in this review.

Where possible, we supplement qualitative findings with quantitative metrics. In particular: (a) we calculate the average reduction in new infections and HIV‐related deaths that was estimated to be possible via a reallocation of funds; (b) we calculate the average increase in treatment coverage that was recommended based on the model findings; and (c) we calculate correlation coefficients between the proportion of new HIV infections acquired by each population (as defined by age/risk/geographical location) and the share of the HIV prevention budget that the model recommended should optimally be targeted at these populations.

We note some limitations of the methods used in this review to synthesize the results of the studies. Given the differences in the inputs that were used for each study and the outputs that were generated, it is challenging to make rigorously quantitative comparative statements; thus, we have kept our comparative analysis general and it should not be considered as a formal meta‐analysis. The 23 studies included here were conducted over a period of several years, and there were various changes to the underlying model, the types of data that were available, and the types of results that were generated during this time. The predictions provided in these studies are limited by the quality of the data and assumptions used to inform them. In numerous settings, there were large uncertainties and/or missing data for key input variables (such as key population sizes, prevalence levels, and/or time trends). Furthermore details of the particular limitations of each study are included in the relevant reports.

## 3 Results and discussion

### 3.1 Estimated epidemiological impact

Across all 23 studies, the modelling results produced by the Optima HIV tool indicated that by reallocating existing funds for HIV, it would be possible to reduce both new HIV infections and HIV‐related deaths. The magnitude of the epidemiological reductions attainable would depend on multiple factors, including the timeframe of consideration, epidemic type and scale, the response profile and level of resourcing available. In 12 studies, the primary objective of analyses (aligned with national strategic plans) was to minimize new infections by 2020; in these studies, the modelling results indicated that an additional 18% (IQR 6% to 29%) reduction in new infections would be attainable by optimally allocating resources (on top of the reductions due to continuing current HIV responses). In the eight studies where the time horizon for minimizing new infections was 2030, an average reduction in new infections of 25% (IQR 4% to 30%) was estimated to be possible through better allocation of resources. (The remaining three studies considered timelines to 2025 or 2010; see Table 2.) Better targeting of resources was also able to further reduce estimated deaths: by 22% (IQR 9% to 28%) on average (across nine studies) by 2020, and by 29% (IQR 7% to 36%) on average (across eight studies) by 2030. Details are summarized in Table 2.

**Table 2:** Summary of results from 23 allocative efficiency studies

Key data							Optimization results under the current budget				Funding required for NSP targets^a^
Country	Year^b^	Epidemic	PLHIV^b^	ART coverage (% of PLHIV)	Budget (US$m)	US$/PLHIV	Programme priority areas	Optimal ART coverage (% of PLHIV)	% reduction in infections	% reduction in deaths	Funds required as a % of current budget
Eastern Europe and Central Asia											
Armenia	2013	Concentrated	3600	65%	4.5	1259	↑ Scale‐up ART, OST, programmes for PWID & FSW – Maintain PMTCT, programmes for prisoners & PWID ↓ Scale‐down GP programmes (SBCC, HTC)	94%	17%^c^	29%^c^	265%
Belarus	2013	Concentrated	35,000	32%	20.5	586	↑ Scale‐up ART, OST, programmes for PWID – Maintain PMTCT, programmes for FSW & MSM ↓ Scale‐down GP programmes (SBCC, HTC)	46%	7%^c^	25%^c^	125%
Bulgaria	2014	Concentrated	6000	21%	8.6	1437	↑ Scale‐up OST, programmes for PWID, MSM & prisoners – Maintain ART, programmes for FSW ↓ Scale‐down GP programmes (SCCC, HTS)	21%	21%^d^	7%^d^	264%
Georgia	2014	Concentrated	8900	32%	14.7	1657	↑ Scale‐up ART, HTC for KPs, programmes for MSM – Maintain programmes for PWID & FSW, OST (60%) ↓ Scale‐down GP programmes (HTC)	59%	16%^d^	36%^d^	140%
Kazakhstan	2013	Concentrated	23,000	22%	34.0	1478	↑ Scale‐up ART, HTC, programmes for PWID & MSM – Maintain PMTCT, programmes for FSW ↓ Scale‐down GP programmes (SBCC, HTC)	30%	6%^c^	22%^c^	137%
Kyrgyz Republic	2013	Concentrated	7500	13%	16.0	2130	↑ Scale‐up ART, HTC, programmes for PWID & MSM – Maintain PMTCT, OST, programmes for FSW	41%	28%^c^	53%^c^	190%
Macedonia	2013	Concentrated	900	22%	6.5	7209	↑ Scale‐up ART, HTS for KPs, programmes for MSM – Maintain programmes for PWID (NSP, OST) & FSW ↓ Scale‐down GP programmes (SBCC)	63%	85%^d^	87%^d^	100%
Moldova	2013	Concentrated	15,000	24%	0.8	51	↑ Scale‐up ART, programmes for FSW, PWID & MSM – Maintain PMTCT ↓ Scale‐down GP programmes (condoms, HTC)	38%	20%^c^	16%^c^	233%
Tajikistan	2013	Concentrated	15,000	10%	14.1	940	↑ Scale‐up ART, all KP programmes – Maintain HTC, PMTCT ↓ Scale‐down Youth, community mobilization, SBCC	15%	5%^c^	Not incl.	Not incl.
Ukraine	2013	Concentrated	210,000	30%	85.2	406	↑ Scale‐up ART, lab monitoring – Maintain all KP programmes, PMTCT ↓ Scale‐down GP programmes (HTC)	41%	3%^c^	9%^c^	Not incl.
Uzbekistan	2011 to 2012	Concentrated	42,000	16%	21.1	502	↑ Scale‐up ART, HTC – Maintain all other prevention ↓ Scale‐down youth programmes	17%	44%^c^	Not incl.	Not incl.
Latin America and the Caribbean											
Argentina	2012	Concentrated	100,000	41%	501.9	5020	– Maintain response	41%	0%^d^	0%^d^	Not incl.
Colombia	2012	Concentrated	130,000	45%	60.0	545	↑ Scale‐up ART, programmes for MSM & homeless ↓ Scale‐down GP programmes (HTC)	53%	28%^d^	24%^d^	Not incl.
Mexico	2011	Concentrated	170,000	52%	432.4	2298.5	↑ Scale‐up ART – Maintain PMTCT ↓ Scale‐down GP programmes	56%	4%^d^	7%^d^	125%
Peru	2014	Concentrated	88,000	57%	91.8	1044	↑ Scale‐up ART – Maintain PMTCT ↓ Scale‐down GP programmes (condoms, SBCC, HTC)	57%	38%^d^	33%^d^	Not incl.
Sub‐Saharan Africa											
Zambia	2012	Mixed	1,100,000	55%	284.2	258	↑ Scale‐up ART, programmes for FSW – Maintain PMTCT ↓ Scale‐down HTC, GP programmes	60%	5%^d^	36%^d^	133%
East Asia and the Pacific											
Indonesia	2012	Mixed	590,000	9%	87.0	147	↑ Scale‐up OST, programmes for PWID, MSM, FSW ↓ Scale‐down GP programmes (condoms, SBCC, HTC)	Not incl.^e^	5%^c^	2%^c^	Not incl.
Vietnam	2012	Concentrated	250,000	Not incl.	136.1	544	↑ Scale‐up HTC, programmes for FSW, MSM ↓ Scale‐down GP programmes, NSP, OST, STI programmes	Not incl.^e^	16%^f^	1%^f^	Not incl.
West and Central Africa											
Cote d'Ivoire	2013	Mixed	470,000	29%	106.0	226	↑ Scale‐up ART, HTC, FSW programmes ↓ Scale‐down GP programmes (condoms, HTC)	32%	5%^c^	6%^c^	283%
Niger	2012	Concentrated	54,000	24%	16.1	298	↑ Scale‐up ART, PMTCT, FSW programmes – Maintain programmes for prisoners, migrants, MSM, mine workers, truckers, OVC, PEP ↓ Scale‐down GP programmes	43%	30%^g^	19%^g^	Not incl.
Senegal	2013	Concentrated	48,000	33%	24.3	505	↑ Scale‐up ART, PMTCT, programmes for FSW & MSM ↓ Scale‐down GP programmes (HTC, SBCC)	50%	31%^c^	28%^c^	Not incl.
Sudan	2013	Concentrated	56,000	6%	12.3	220	↑ Scale‐up ART, programmes for FSW & clients & MSM ↓ Scale‐down GP programmes	12%	36%^c^	Not incl.	134%
Togo	2014	Mixed	110,000	31%	20.1	183	– Maintain response	31%	0%^g^	0%^g^	155%
**Averages**											
				30%		1285		42%	18% to 2020 25% to 2030	22% to 2020 29% to 2030	176%

ART, antiretroviral therapy; OST, opiate substitution therapy; PWID, people who inject drugs; FSW, female sex workers; PMTCT, prevention of mother‐to‐child transmission; MSM, men who have sex with men; GP, general population; SBCC, social and behaviour change communication; HTC, HIV testing and counselling; OVC, orphans and vulnerable children; KP, key population; VL, viral load; PEP, post‐exposure prophylaxis; Not incl., indicator not requested for this study.

a Percentage increase over the total expenditure at the last NASA that would be required to meet the National Strategic Plan (NSP) targets, assuming that funds were optimally allocated.

b Year for which latest National AIDS Spending Accounts were available at the time study was conducted, and estimate of the number of PLHIV in that year as published in the country reports.

c Percentage reduction in cumulative infections/deaths over the years until 2020 that could be obtained via optimally allocating resources.

d Percentage reduction in cumulative infections/deaths over the years until 2030 that could be obtained via optimally allocating resources.

e In Vietnam, and Indonesia, ART was not considered as part of the pool of funding available for reallocation but rather as required resources earmarked as an essential expense. Therefore, we did not estimate optimal coverage levels for these two countries.

f Percentage reduction in cumulative infections/deaths over 2006 to 2010 that could be obtained via optimally allocating resources.

g Percentage reduction in cumulative infections/deaths over the years until 2025 that could be obtained via optimally allocating resources.

### 3.2 Increased allocations to treatment

If no increases in the overall HIV budget are expected, the analyses recommended increasing the share of HIV budgets allocated to ART from 49% to 64% on average, which would in turn increase estimated average national ART coverage from 30% to 42% as a percentage of all PLHIV (Table 2).

The model's recommendations to expand treatment had important but varied consequences for the role of HIV testing and counselling (HTC) programmes. In 14 countries, there was known to be a large pool of people who had already been diagnosed but were not on treatment. In these cases, the modelling results indicated that it would be better to increase funding to ART programmes first (including programmes linking and retaining people to care), and that testing programmes should not be scaled up until those already diagnosed had been initiated on ART. This was consistently the case, even when testing programmes were delivered via high‐yield or low‐cost service modalities.

### 3.3 Increased allocations to the populations with the highest incidence

**Figure 1:**
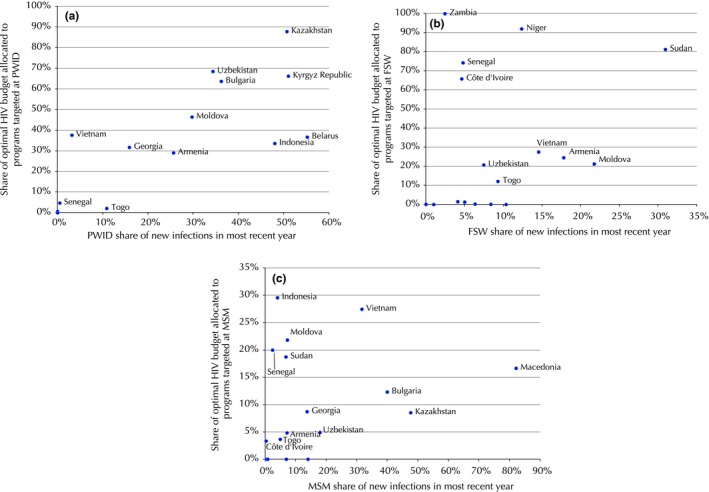
The relationship between the share of infections in a particular population/district, and the share of the HIV budget for prevention programmes. Results pertain to the year for which latest National AIDS Spending Accounts were available at the time study was conducted – these years are presented in Table 2. The share of infections by sub‐population was not available for Peru, Mexico, Colombia, Argentina, Tajikistan or Ukraine. **(a)** PWID across 17 countries, **(b)** FSW across 17 countries, **(c)** MSM across 17 countries.

There was a marked correlation between the share of new HIV infections acquired by each population and the share of the HIV prevention budget that the model recommended should be targeted to them. The correlation coefficient was particularly high (0.77) for programmes targeted at PWID (Figure 1a). The overall correlation coefficient was lower (0.43) in the case of programmes for FSW, but this was strongly influenced by the three countries from Western and Central Africa in which rates of indirect sex work amongst the general female population are high, so programmes targeted to the general female population are likely to be effective proxies. After removing these countries, we found a correlation coefficient of 0.88 (Figure 1b). However, we found that the correlation coefficient was lower for programmes targeted at MSM (0.20; Figure 1c).

Almost all countries experience geographical variation in both the severity of the HIV epidemic and in the costs of delivering the HIV response. Often – especially in concentrated epidemics – there is significant overlap between the geographical distribution of the HIV epidemic and the geographical distribution of the key affected populations, and as a result it may be possible to ensure that the HIV response is targeted at the right places simply by targeting the key populations, where such populations can be safely found and supported with programmes. However, in areas where there is significant stigma and discrimination or where some key population behaviour is illegal, geographic targeting as a proxy for key population targeting might be needed in order to ensure the safety of programme staff and the key populations themselves. When considering mixed and generalized epidemics, the geographical distribution of the HIV epidemic among the general population must also be taken into account.

### 3.4 Targeting the right delivery approaches to maximize coverage

The question of how best to target the HIV response to the appropriate populations and geographic areas is crucial, but it must be considered alongside the equally important question of how to deliver these HIV services at the highest possible quality and the lowest feasible cost in ways that will reach a wide variety of the intended populations. In general, information on the heterogeneity of the costs and the impact of delivering HIV services both within and across countries is scarce, and information on the determinants of this variation is even scarcer. Across all 23 studies, significant attention was given to the question of how the overall HIV response could be improved by lowering costs while maintaining or improving service delivery quality and modality to ensure the highest coverage. In general, the recommendation was for countries to rigorously review the costs of delivering their HIV responses with a focus on both the unit costs of delivering core HIV services and on the costs associated with management, human resources, administration, enablers, support and synergies.

With regard to unit costs of service delivery, we found large heterogeneities both between countries and within countries. Across the 11 studies completed in the Eastern Europe & Central Asia region, there was significant variation in the unit costs of ART, FSW programmes, MSM programmes, opiate substitution therapy programmes, and needle‐syringe programmes 45. A separate study found large variation in the unit costs of delivering needle‐syringe programmes, opiate substitution therapy, and ART across 52 sites in three oblasts in Ukraine 46. It can be difficult to distinguish intrinsic heterogeneity from inefficiency, but benchmarking exercises are the first step in identifying potential mechanisms for streamlined service delivery.

It is also difficult to make a cross‐country comparison of expenditure on management and other supporting programmes, as methods for accounting for these costs vary significantly across countries and not all costs were included in all studies. It was not within the scope of these studies to identify ways for strategic purchasing (or commissioning) for reducing cost components of HIV responses, but many of the stakeholders involved in the studies indicated that cost reductions were desired and potentially feasible.

### 3.5 The optimal programme mix varies by resource availability

In 16 of the 23 studies, the optimal distribution of the total HIV budget under different funding constraints was estimated. Across these studies, it was found that when very little money is available, the optimal strategy is to focus on funding fewer programmes in order to take advantage of economies of scale, rather than continuing to fund the full mix of programmes at lower levels. As more resources become available, the next most cost‐effective programmes should be introduced and then scaled up. This was consistently the case across all 16 studies that contained this analysis (Table 2), and has been explored in greater detail in a separate publication 47.

### 3.6 More resources are required to achieve national targets

In 13 of the 23 studies, the minimal level of investment required to achieve the epidemiological targets described in the country's national strategic plan was estimated. In all but one case (Macedonia), the amount being invested in the HIV response at the time that the study was conducted was estimated to be insufficient. The modelling results indicated that budget increases up to 185% would be required to attain the targets within the strategic plan timeframes (Table 2). This is consistent with estimates published elsewhere of the resources required to achieve global HIV targets 48. Note that these resource estimates pertain to the targets contained in the national strategic plan that was in place at the time that the study was conducted, which may since have changed. A detailed description of the particular targets is contained in each of the reports.

Knowledge of the funding environment and the likely amount of resources that will be made available for HIV is an essential component of planning an effective HIV response. The majority of countries around the world have set ambitious national targets for HIV reduction, yet have not invested or acquired close to the level of resources for direct HIV programmes necessary to realistically achieve these targets, even if their resources were invested in the best possible mix of programmes. It may be possible to free up more funds for core HIV services by improving the overall technical efficiency of the HIV response – for example, via the integration of HIV services into primary care, or by leveraging regional‐level negotiating power to bring down drug procurement costs – but even after exploiting all possible gains from technical and allocative efficiency improvements, it is almost certainly still the case that additional funds will be required 48.

### 3.7 Adoption of model recommendations

**Figure 2:**
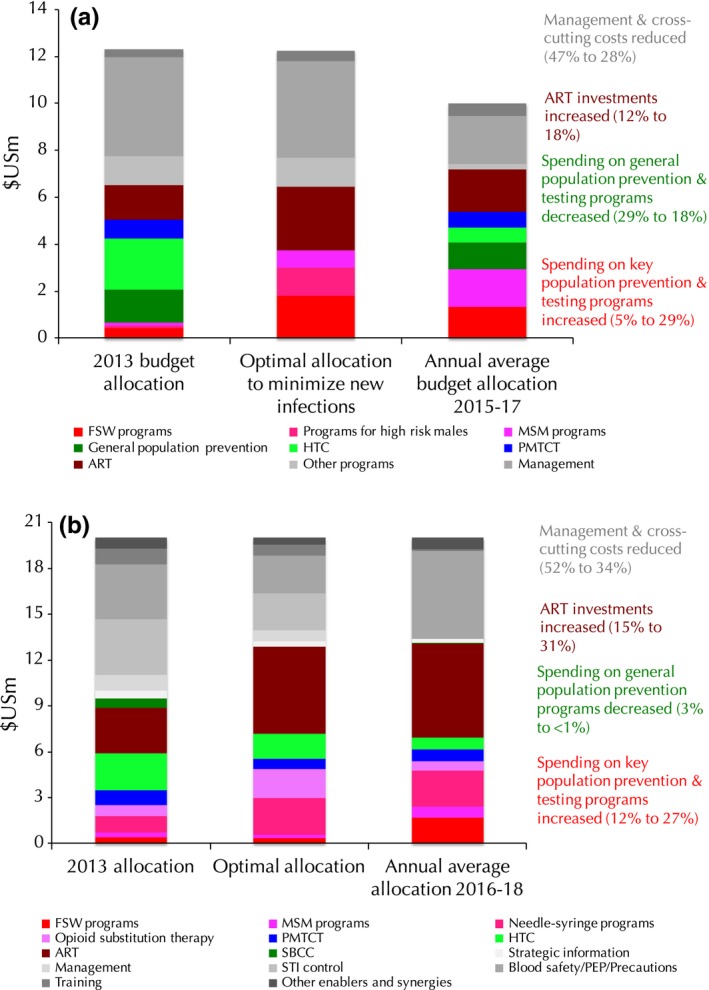
Allocations of HIV budgets prior to Optima HIV study (left bars), the mathematically optimal allocation recommended by the Optima HIV analysis (middle bars) and the allocation that was adopted by the country after the budgeting process was complete (right bars). **(a)** Sudan **(b)** Belarus. Note that in Sudan, the total budget envelope was decreased from US$12.3 m to US$9.9 m.

Allocative efficiency studies are most useful when conducted prior to the budget‐ or target‐setting process, so that they can help inform health‐related targets and determine the funding envelopes and allocations commensurate with these targets. The studies conducted in Sudan and Belarus represent two examples from the suite of studies considered in this review where the timing meant that the studies’ recommendations could be taken into account in the budget‐setting process, and both countries ultimately shifted their HIV budget allocations closer towards what the optimization analyses recommended (Figure 2). Most other countries have used the results of the Optima studies to inform their planning processes, resource allocations or programmatic priorities. We hope to generate evidence of these examples where modelling has been useful to improve disease control strategies.

Analyses such as these are just one step in the process of bringing about optimal resource allocation and maximum health outcomes; the real challenge lies in mobilizing funding and potentially changing the nature or type of programmes that policymakers implement. This can be challenging due to the multitude of funding sources and the large proportion of HIV funding that is dictated by external funding agendas and allocation criteria. Policy recommendations will be most useful when they are accompanied with an operational plan, supplemented with technical support, which sets out a clear pathway and implementation details. Whilst priority setting is a difficult process, it will become increasingly important as countries move toward universal health coverage and will need to make decisions based on objectives, health system users and cost‐effectiveness.

## 4 Conclusions

Our study collates the findings of 23 country analyses that modelled how to optimally allocate the distribution of funds across HIV programme areas in real‐world budget allocation decision‐making. According to the results produced in these country studies, it would be possible to achieve substantial (5% to 30%) reductions in infections and deaths through better targeting of existing resources. The findings also underscore the benefits of additional resources targeted towards HIV prevention and care. Having tools such as Optima HIV to assist country decision‐makers and their partners has value in improving the HIV planning and budgeting process.

## Competing interests

The authors declare no competing interests.

## Authors’ contributions

RMS, LG and DPW wrote the manuscript. Analyses were carried out by HH‐B, JS, JP‐G, LG, OK, JE, ZB, SLK, IR, DJK, AJS, JP, SAH, KLG, RTG, XFY, RM‐H, CJB, NF‐H, EM, ZS, NC, RMS and CCK. Guidance was provided by MG, EM, NC and ZS. The Optima HIV software (used to conduct analyses) was primarily developed by CCK, RMS, DJK, AJS, RTG and DPW, with substantial technical inputs from CJB, NF‐H, DJW, MG, EM, EM, NC and ZS.

## Supporting information


**Table S1:** Full list of Optima HIV studies.
