# Supplementary material for: How should HIV resources be allocated? Lessons learnt from applying Optima HIV in 23 countries
Source: J Int AIDS Soc. 2018 Apr 13;21(4):e25097. doi: 10.1002/jia2.25097 (PMC5898225; doi:10.1002/jia2.25097)
Supplement: Supplementary file 1 — Table S1: Full list of Optima HIV studies. [file JIA2-21-e25097-s001.docx]

Supplementary material

How should HIV resources be allocated? Lessons learnt from applying Optima HIV in 23 countries

# Table S1. Full list of Optima HIV studies

| *Region* | *Country* | *Purpose of study* | Completion year | Included | Comments |
| --- | --- | --- | --- | --- | --- |
| Asia-Pacific | Cambodia | Research | 2009 | N | Excluded as not conducted for government |
| Asia-Pacific | Cambodia | Research | 2016 | N | Excluded as not conducted for government |
| Asia-Pacific | China | Research | 2016 | N | Excluded as not conducted for government |
| Asia-Pacific | India | Research | 2016 | N | Excluded as not conducted for government |
| Asia-Pacific | Indonesia | Government | 2011 | N | Excluded because a more recent study was available |
| Asia-Pacific | Indonesia | Government | 2012 | Y |  |
| Asia-Pacific | Indonesia | Government | 2015 | N | Excluded as study did not look at optimizing resource allocations |
| Asia-Pacific | Indonesia | WHO | 2016 | N | Excluded as study did not look at optimizing resource allocations |
| Asia-Pacific | Myanmar | Government | 2017 | N | Excluded as study was confidential to government |
| Asia-Pacific | Nepal | Research | 2016 | N | Excluded as not conducted for government |
| Asia-Pacific | Papua New Guinea | Government | 2011 | N | Excluded as study did not look at optimizing resource allocations |
| Asia-Pacific | Papua New Guinea | Research | 2009 | N | Excluded as not conducted for government |
| Asia-Pacific | Papua New Guinea | Research | 2016 | N | Excluded as not conducted for government |
| Asia-Pacific | Philippines | World Bank | 2013 | N | Excluded as study did not look at optimizing resource allocations |
| Asia-Pacific | Thailand | Government | 2015 | N | Excluded as study only included Bangkok |
| Asia-Pacific | Thailand | WHO | 2016 | N | Excluded as study did not look at optimizing resource allocations |
| Asia-Pacific | Vietnam | Donors | 2015 | N | Excluded as study did not look at optimizing resource allocations |
| Asia-Pacific | Vietnam | Government | 2011 | N | Excluded because a more recent study was available |
| Asia-Pacific | Vietnam | Government | 2012 | Y |  |
| Asia-Pacific | Vietnam | Research | 2014 | N | Excluded as not conducted for government |
| EECA | Armenia | Government | 2014 | N | Excluded because a more recent study was available |
| EECA | Armenia | Government | 2016 | Y |  |
| EECA | Armenia | UNAIDS | 2012 | N | Excluded as not conducted for government |
| EECA | Belarus | Government | 2013 | N | Excluded because a more recent study was available |
| EECA | Belarus | Government | 2016 | Y |  |
| EECA | Belarus | UNAIDS | 2012 | N | Excluded as not conducted for government |
| EECA | Bulgaria | Government | 2016 | Y |  |
| EECA | Estonia | UNAIDS | 2012 | N | Excluded as not conducted for government |
| EECA | Georgia | Government | 2015 | Y |  |
| EECA | Georgia | UNAIDS | 2012 | N | Excluded as not conducted for government |
| EECA | Kazakhstan | Government | 2016 | Y |  |
| EECA | Kazakhstan | UNAIDS | 2012 | N | Excluded as not conducted for government |
| EECA | Kyrgyzstan | Government | 2016 | Y |  |
| EECA | Macedonia | Government | 2016 | Y |  |
| EECA | Moldova | Government | 2016 | Y |  |
| EECA | Moldova | UNAIDS | 2012 | N | Excluded as not conducted for government |
| EECA | Russia | Research | 2016 | N | Excluded as not conducted for government |
| EECA | Tajikistan | Government | 2014 | Y |  |
| EECA | Tajikistan | Research | 2016 | N | Excluded as not conducted for government |
| EECA | Tajikistan | UNAIDS | 2012 | N | Excluded as not conducted for government |
| EECA | Ukraine | Government | 2014 | Y |  |
| EECA | Ukraine | Research | 2016 | N | Excluded as not conducted for government |
| EECA | Ukraine | UNAIDS | 2012 | N | Excluded as not conducted for government |
| EECA | Ukraine | Evaluation | 2016 | N | Excluded as study did not look at optimizing resource allocations |
| EECA | Uzbekistan | Government | 2015 | Y |  |
| EECA | Uzbekistan | Research | 2016 | N | Excluded as not conducted for government |
| High-income | Australia | Government | 2007-2014 | N | Excluded as study did not look at optimizing resource allocations |
| High-income | Canada | Government | In progress | N | Excluded as study did not look at optimizing resource allocations and was confined to a single province |
| LAC | Argentina | Government | 2016 | Y |  |
| LAC | Brazil | Research | 2016 | N | Excluded as not conducted for government |
| LAC | Colombia | Government | 2016 | Y |  |
| LAC | Mexico | Government | 2016 | Y |  |
| LAC | Peru | Government | 2016 | Y |  |
| MENA | Cote d'Ivoire | Government | Final stages | Y |  |
| MENA | Iran | Research | In progress | N | Excluded as not conducted for government |
| MENA | Niger | Government | 2014 | Y |  |
| MENA | Senegal | Government | Final stages | Y |  |
| MENA | Senegal | Research | 2016 | N | Excluded as not conducted for government |
| MENA | Sudan | Government | 2014 | Y |  |
| MENA | Togo | Government | Final stages | Y |  |
| SSA | Cameroon | Research | 2016 | N | Excluded as not conducted for government |
| SSA | DR Congo | Research | 2016 | N | Excluded as not conducted for government |
| SSA | Ethiopia | Research | 2016 | N | Excluded as not conducted for government |
| SSA | Kenya | Research | 2016 | N | Excluded as not conducted for government |
| SSA | Malawi | Government | In progress | N | Excluded as study is incomplete |
| SSA | Mozambique | Research | 2016 | N | Excluded as not conducted for government |
| SSA | Nigeria | Research | 2016 | N | Excluded as not conducted for government |
| SSA | South Africa | Research | 2005-2008 | N | Excluded as not conducted for government |
| SSA | South Africa - Johannesburg | Government | 2016 | N | Excluded as confined to a single city |
| SSA | South Africa | Research | 2016 | N | Excluded as not conducted for government |
| SSA | Swaziland | Government | 2014 | N | Excluded as study was confidential to government |
| SSA | Tanzania | Research | 2016 | N | Excluded as not conducted for government |
| SSA | Uganda | Research | 2016 | N | Excluded as not conducted for government |
| SSA | Zambia | Government | 2015 | Y |  |
| SSA | Zambia | Research | 2015 | N | Excluded as not conducted for government |
| SSA | Zimbabwe | Research | 2016 | N | Excluded as not conducted for government |
